# A machine learning approach to identify the universality of solitary perturbations accompanying boundary bursts in magnetized toroidal plasmas

**DOI:** 10.1038/s41598-021-83192-2

**Published:** 2021-02-11

**Authors:** J. E. Lee, P. H. Seo, J. G. Bak, G. S. Yun

**Affiliations:** 1 Pohang University of Science and Technology, Pohang, 37673 Korea; 2 National Fusion Research Institute, Daejeon, 34133 Korea

**Keywords:** Magnetically confined plasmas, Computer science

## Abstract

Experimental observations assisted by 2-D imaging diagnostics on the KSTAR tokamak show that a solitary perturbation (SP) emerges prior to a boundary burst of magnetized toroidal plasmas, which puts forward SP as a potential candidate for the burst trigger. We have constructed a machine learning (ML) model based on a convolutional deep neural network architecture for a statistical study to identify the SP as a boundary burst trigger. The ML model takes sequential signals detected from 19 toroidal Mirnov coils as input and predicts whether each temporal frame corresponds to an SP. We trained the network in a supervised manner on a training set consisting of real signals with manually annotated SP locations and synthetic burst signals. The trained model achieves high performances in various metrics on a test data set. We also demonstrated the reliability of the model by visualizing the discriminative parts of the input signals that the model recognizes. Finally, we applied the trained model to new data from KSTAR experiments, which were never seen during training, and confirmed that the large burst at the plasma boundary that can fatally damage the fusion device always involves the emergence of SP. This result suggests that the SP is a key to understanding and controlling of the boundary burst in magnetized toroidal plasmas.

## Introduction

Nuclear fusion, which generates solar energy, has been considered as one of the promising alternative energy sources to meet the increasing global energy demand by providing an environmentally friendly and almost limitless source of energy^1^. To realize a sustained nuclear fusion on Earth, the torus-shaped fusion device called tokamak uses axisymmetric strong magnetic fields to confine a high-temperature plasma^2,3^. A tokamak plasma with a particular magnetic field configuration and a high heating power exceeding a threshold can attain a highly confined state (H-mode)^4,5^. An H-mode plasma has a transport barrier in the boundary region due to increased radial electric field shear that reduces heat and particle transport^6–8^. However, the improved confinement comes with an adverse side effect. The transport barrier region becomes susceptible to fluid instabilities (due to increasing pressure gradient, current density, and flow) and eventually collapses, resulting in a rapid radially outward burst of charged particles and heat from the plasma to the outside. The boundary burst starts from a formation of localized channels connecting the inside and outside of the plasma (presumably by magnetic reconnection)^9–12^ and spreads quickly over the entire plasma surface, reminiscent of a bursting balloon pricked by a needle. The repetitive formation and destruction of the transport barrier is a non-desirable characteristic feature of the H-mode because the high flux of energy and particles released during the burst can severely damage the plasma-facing components of the tokamak^13^. Therefore, controlling the boundary burst based on an accurate understanding of the underlying mechanism is essential for the safe operation of fusion devices. The boundary burst has been commonly interpreted in relation to edge localized modes (ELMs), which are a class of magnetohydrodynamic (MHD) instabilities driven by steep pressure gradient and local current density in the boundary region^14–17^. However, it remains uncertain whether the development of ELM is a sufficient condition for the boundary burst. In particular, the detailed ELM dynamics visualized by electron cyclotron emission (ECE) imaging diagnostics on the Korea superconducting tokamak advanced research (KSTAR) device^9,10^ suggested that ELM is not a direct trigger of the burst. The 2-D ECE images showed that an ELM lasts up to a few ms without burst and the mode suddenly disappears prior to the boundary burst^9,10,18^, indicating that the burst is triggered in the absence of ELM.

We have recently reported a new type of perturbation with the spatially solitary structure that appears within hundreds of μs before the onset of the boundary burst in the KSTAR device^12,19,20^. The existence of solitary perturbation (SP) was confirmed by the three independent ECE imaging (ECEI) systems that are capable of quasi-3D visualization of ECE radiation temperature ($T_{\rm{ece}}$) fluctuations^21,22^. The structure and dynamics of SPs measured by the ECEI systems were consistent with the observations by a toroidal array of 19 Mirnov coils (MCs) that can provide information on the global structure of the poloidal magnetic field fluctuations ($\delta B_{\theta }$)^23^. The SP structure localized in the poloidal direction is clearly distinguished from the structure of the ELM, which is a poloidal array of filaments with a finite wavelength. Furthermore, the SP has a different rotational speed, and a smaller pitch angle compared to the ELM preceding the SP. In other fusion devices, strong magnetic perturbations, distinct from the ELM during the inter-burst period, have been also observed before the abrupt onset of the burst^24–26^. Moreover, many simulations have reported that a new mode can emerge via nonlinear coupling between dominant ELMs before the onset of the burst^27–29^. These results support the idea that the emergence of SP, not ELM, triggers the burst phase.

A statistical study on extensive data would be a prerequisite before ascertaining the SP as the burst trigger. On the contrary, the reported cases of SP are scarce so far because the proximity of the SP signals to the boundary burst signals of overwhelming intensity makes difficult the manual identification of SP. To overcome the difficulty, we have developed an automatic SP identification model based on deep neural network^30^, which is specialized for meaningful feature extraction from the high-dimensional data. The input to the model is time-series data of 19 toroidal Mirnov coils in the KSTAR, and the output is the prediction of SP for each temporal frame. We trained the network in a supervised manner on a training data set. Note that we included synthetic burst data in the training data set, which imitate boundary burst without SPs, to avoid false recognition of the network due to the near-concurrency of the SP and the burst. We quantitatively confirmed the high performance of the developed model with various metrics. We also used the gradient-based network visualization technique to qualitatively verify that the SP patterns greatly influence the predictions of the model. Finally, we performed a statistical analysis on the concurrence of boundary burst and SP using the developed SP identification model.

## Results

### Brief description of solitary perturbation

**Figure 1:**
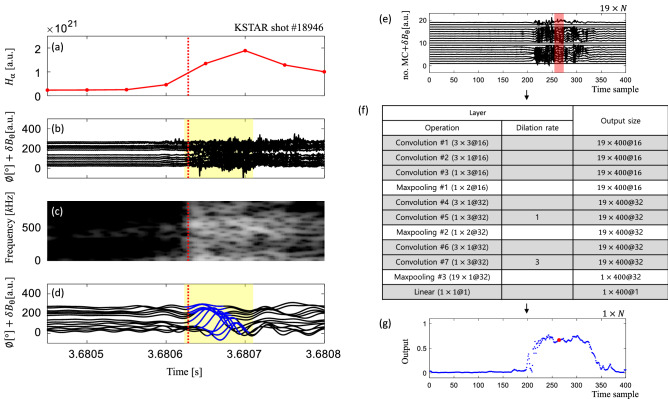
An example of solitary perturbation (KSTAR shot #18946) and network architecture for the SP identification model. (**a**) $H_{\alpha }$ signal, (**b**) 19 raw toroidal MC signals, (**c**) spectrogram of the time-series data for the 11th toroidal MC, and (**d**) band-pass filtered MC signals (5–30 kHz) of the example. SP is highlighted in blue color. Red vertical dotted line and yellow box in (**a**)–(**d**) respectively indicate the start time of the boundary burst and the duration of the SP (~ 90 μs). (**e**) network input: 19 toroidal MC signals with 1 MHz sampling frequency, (**f**) network architecture, and (**g**) network output: SP probability corresponding to each temporal frame. One output frame, indicated by a red dot in (**g**), is affected by the input region of 19 coils and 19 time samples (corresponding to 19 μs) highlighted in the red box in (**e**).

The boundary burst involves high particle flux and rapid changes in the magnetic fields, making it easy to detect. The former leads to increased $H_{\alpha }$ emission, and the latter results in high-amplitude MC signals with broadband spectrum (Fig. 1). Low-pass filtering (< 50 kHz) of the toroidal MC signals reveals a spatially localized (solitary) perturbation of high amplitude in proximity to the burst, as highlighted in blue color in Fig. 1d, which is not easily discernible in raw MC signals.

The solitary perturbation survives a few tens of μs to hundreds of μs while propagating in the toroidal direction in the laboratory frame without a noticeable change in shape^19^. The red dotted lines in Fig. 1 represent the start time of the burst in an example case (KSTAR shot #18946). Here, we determine the start time by the rapid increase in the sum of all the toroidal MC signals (see supplementary Fig. S1 for detail) because the MC signals capture directly the magnetic field perturbations caused by the burst. Another reason for determining the start timing of the burst based on MC signals instead of $H_{\alpha }$ signals is that the time resolution of $H_{\alpha }$ detector is not sufficient to resolve the SP and the burst occurring with a time difference of tens of μs. The SP duration in this example is ~ 90 μs, as highlighted in yellow in Fig. 1. Our observation indicates that the SP develops near the onset of boundary burst and persists for a while during the burst.

### Development of the identification model of solitary perturbation

The bottleneck of a large-scale statistical analysis on the co-occurrence of SP and boundary burst is the identification problem: the existence of an SP is not evident in raw MC signals. In this work, we have developed a machine learning (ML) model based on a convolutional neural network (CNN) for automatic identification of the existence of SP in an input MC signal. Detailed explanations of CNN and important notations used in the developed network are covered the section on ‘Convolutional neural network’ in Methods .

Our network takes a set of 19 toroidal raw MC signals $X \in {{\mathbb{R}}^{19\times N}}$ captured with 1 MHz sampling frequency (Fig. 1e), and predicts a sequence of probabilities ${\mathbf{y}}\in {\mathbb{R}}^{N}$ where *N* is the number of temporal frames (Fig. 1g). Note that ${\mathbf{x}}_t \in {\mathbb{R}}^{19}$ and $y_t \in {\mathbb{R}}$ respectively represent a vector consisting of input signals captured from 19 toroidal MCs and an output probability indicating whether an SP is present at time *t*. The network consists of 11 layers, as depicted in Fig. 1f: seven 2D convolutional layers with rectified linear unit (ReLU) activation, three max-pooling layers, and one linear layer with a sigmoid activation function. As it is required to identify the temporally shifted solitary patterns across the MCs for predicting SPs, we adopted 2D convolution operations that capture local context patterns within the input matrix *X*. Each layer configuration is denoted by $n \times m @ l$, where *n* and *m* are the toroidal coil dimension and the temporal dimension of a kernel, respectively, and *l* is the number of output channels. At each layer, we apply a circular padding mechanism in the toroidal coil dimension to take into account the toroidal installation of the MCs. In contrast, we apply zero paddings to the temporal dimension to keep the number of temporal frames. If we set the stride of every operation to one to maintain the original resolution of the input signals, it results in highly overlapped receptive fields between adjacent hidden activations in feature maps. To avoid this issue and allow a larger receptive field size in the final feature map, we use dilated convolutions. At last, we perform a global max-pooling along with the toroidal coil dimension and feed it to a linear classifier with a sigmoid output to predict the final probability $y_t$ at each temporal frame. Each output value $y_t$ is computed from a $19\times 19$ input region: the region covers all 19 MCs and 19 consecutive temporal frames corresponding to 19 μs centered from time *t* as depicted by a red box in Fig. 1e.

We trained our network in a supervised manner, using data collected from KSTAR discharges from 2015 to 2017. We prepared a training data set by manually annotating SPs at each time frame by a trained expert. The training set consists of 100 positive and 20 negative examples. Each example is 400 μs long, containing 400 frames of the 19-channel MC signals. Note that all positive examples contain both the boundary burst and the SP, whereas the negative ones contain neither of them. The boundary bursts of various intensities are included in the positive examples. Each positive example is a sequence of data sampled from 200 μs prior to the start of burst. Each negative example is a sequence of data sampled from an inter-burst period that is at least 500 μs distant from the emergence of an SP. In addition, we added 20 synthetic burst signals to the training set, which imitates a boundary burst without an SP, in order to prevent the network from falsely recognizing the relatively large-amplitude burst as SP due to their consistent co-occurrences. The boundary burst is characterized by strong MC signals with a broadband spectrum, presumably because of random transport of particles and complex changes of magnetic topology in the plasma boundary. In order to imitate such characteristics, the synthetic burst signals are generated by multiplying white noise onto the envelopes of the real MC signals in the burst period. A detailed comparison between real and synthetic burst signals is shown in Fig. S2. Our final model is an ensemble of three independently trained models with different initializations and random seeds on the same training data set. All hyper-parameters in the algorithm are selected by cross-validation in the training set.

### Performance of the developed model

**Figure 2:**
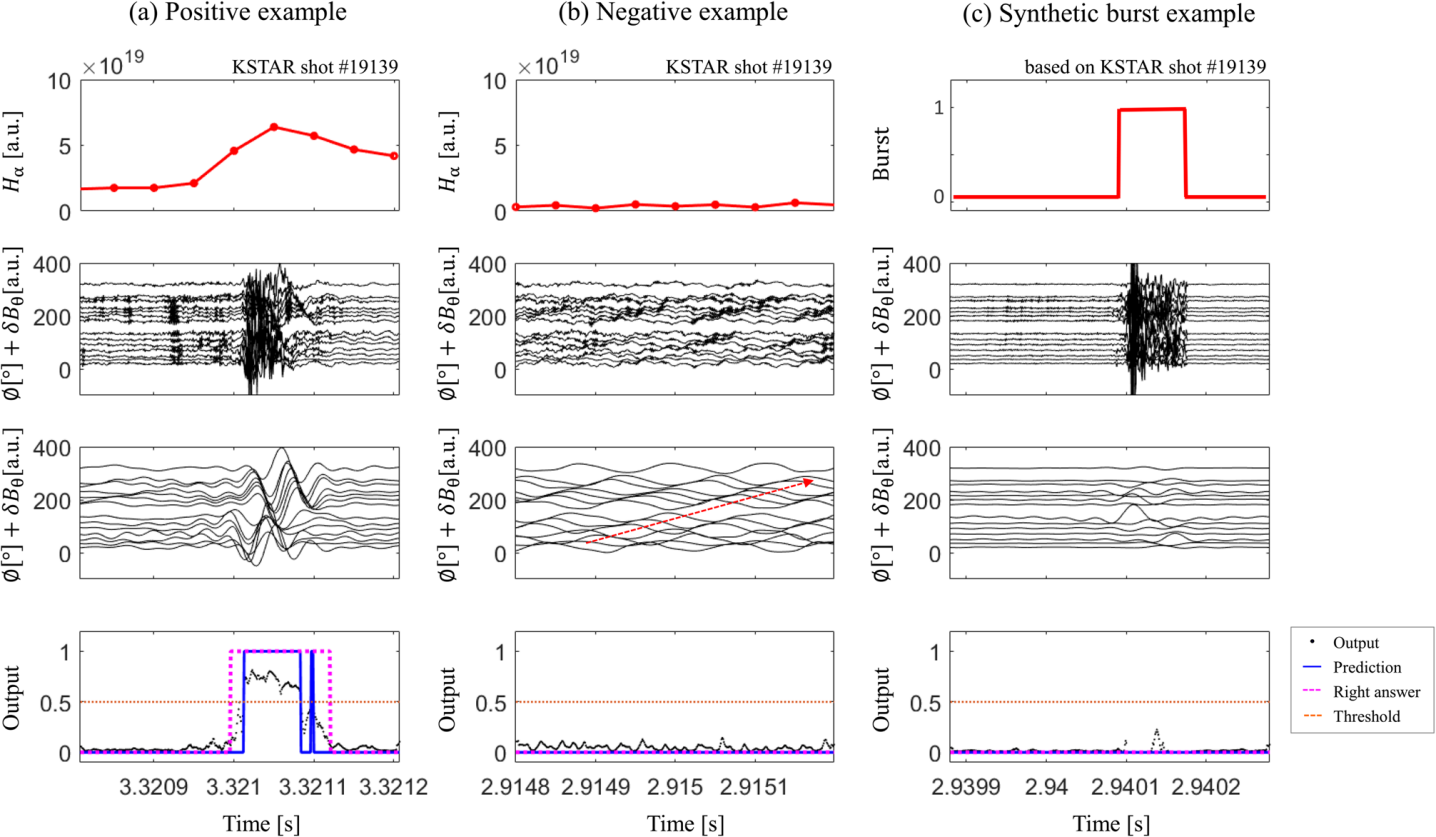
Representative test results of the final ML model. The first row is the $H_{\alpha }$ signal, the second row is the raw MC signals the third row is the band-pass filtered MC signal, and the last row is the output probability of the final model for (**a**) positive example, (**b**) negative example, and (**c**) synthetic burst example. Here, the first row in the synthetic burst example represents the burst period used to create the synthetic burst data instead of $H_{\alpha }$ signal. The black dot, blue line, magenta dotted line and orange dotted line in the last row respectively indicate output probability, prediction of SP existence, right answer of SP occurrence and the threshold of output to determine the presence or absence of SP. The ELM signal during the inter-burst period is shown in the filtered MC data in (**b**). The red dashed arrow indicates the toroidal flow direction of the ELM.

We collected the test data set in a total of 50 sequences, including 26 positive and 12 negative examples in addition to 12 synthetic ones, using the identical method as in the training data collection. Note that the test data set is sampled at discharges separate from the KSTAR discharges in the training data set. We applied our final trained model on the test data set. The representative examples of the test results are demonstrated in Fig. 2: $H_{\alpha }$ signal, raw toroidal MC signals, the MC signals in the frequency range of 3–50 kHz, and outputs of the model are provided for (a) positive example, (b) negative example, and (c) synthetic burst example. Note that the synthetic burst example provides a boundary burst period time used to generate the synthetic data instead of $H_{\alpha }$ signal. We confirmed that the model produces a high output score for the input region with the SP pattern in the positive examples, while the model produces a low output score for the negative and synthetic burst examples. Some negative examples include ELM signals shifted in the toroidal direction (Fig. 2b). Note that our model does not generate a high score because the MC signal contains either a low-frequency toroidal shifted pattern or a relatively strong amplitude.

We evaluated our final trained model using three metrics: (per-frame) accuracy, average precision, and per-sequence accuracy. Accuracy measures the proportion of the correct prediction per frame. However, the accuracy is heavily dependent on the choice of the threshold value, and it may not be an adequate indicator for a good model because of the imbalance between non-SP frames and SP frames in our data. For example, a trivial model that predicts a non-SP for every frame would achieve high accuracy ($82.5 \,\%$) due to the data imbalance. Therefore, we also introduce average precision (AP), which is widely used to measure the performance of the model independent of the threshold value. AP is a mean precision over all possible threshold values weighted by recall. A detailed description of AP is provided in ‘Average precision’ of Methods. Another metric, per-sequence accuracy, measures the quality of the prediction about the presence of an SP within the whole input sequence. For each input sequence, we post-process the output sequence to obtain a single SP prediction value $y_{\rm{s}} \in {\mathbb{R}}$ as follows: 1) Mark the beginning point of an SP where there are three consecutive positive predictions. 2) Accumulate the predicted probabilities for the range of 100 μs centered at the beginning point. We choose that there exists an SP if $y_{\rm{s}}$ exceeds 25. Note that all the parameters in the process are determined by cross-validation in the training set. We use this metric to statistically analyze the co-occurrence of boundary burst and SP.

Our final model achieves $91.5\,\%$ of per-frame accuracy on the test data set with the score threshold of 0.5. Admitting the limitations of the accuracy with an imbalanced data set, it still indicates that our final model performs significantly better than the trivial baseline model ($82.5\,\%$) that always returns negative outputs. Our model achieves AP of $88.5\,\%$, showing reliable per-frame prediction quality. This high AP score indicates that we can maintain both high precision and recall with a proper threshold. Finally, we measured the per-sequence accuracy of the trained model. Our trained model correctly predicts all 50 sequences achieving $100\,\%$ of per-sequence accuracy in the test set.

### Qualitative validation by visualization

**Figure 3:**
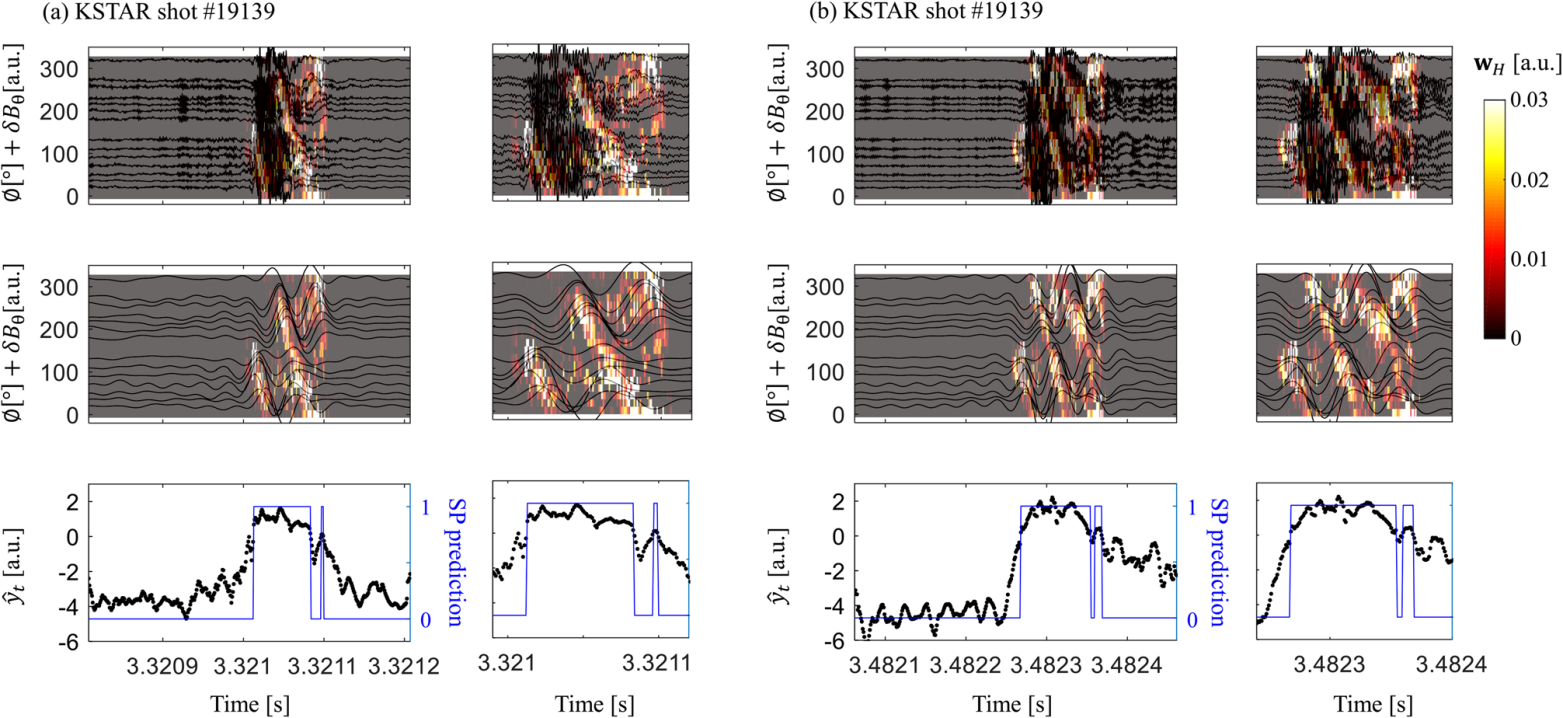
Validation of the final model using a gradient-based visualization technique. Heatmap of ${\mathbf{w}}_H$ is overlaid on the MC raw signals (first row) and band-pass filtered MC signals (second row) for different sequences, (**a**) and (**b**). Third row represents the pre-sigmoid activation of the output ${\hat{y}}_t$ (black dot) and prediction of SP existence (blue line). The figures on the right in (**a**) and (**b**) are enlarged figures of the area of interest in each example. The heatmap ${\mathbf{w}}_H$ shows the influential part of the input area on the positive outputs. The heatmaps were generated using Matlab version R2018a (https://www.mathworks.com/)^31^.

We examine whether our final model identifies SPs based on the shifted solitary patterns as a trained expert would do, to exclude the possibility of simple overfitting to the training set. We do this using a gradient-based visualization technique that is widely adopted to highlight influential parts of input in predicting a certain output^32^. To use this technique, we approximate our network, which is a highly non-linear function, with a linear function in the vicinity of a given input $X_0$ by the first-order Taylor expansion:

1 $$\begin{aligned} {\hat{y}}_t \approx {\mathbf{w}}_t^{\mathbf{T}}{\mathbf{r}}+b_t \qquad {\rm{where}}\quad {\mathbf{w}}_t=\left. \frac{\partial {\hat{y}}_t}{\partial {\mathbf{r}}}\right| _{{\mathbf{r}}_0} \end{aligned}$$

Here, ${\hat{y}}_t$ is an input to the last sigmoid function which yields $y_t$, *i.e.*, $y_t={\rm{sigmoid}}({\hat{y}}_t)$, ${\mathbf{r}} \in {\mathbb{R}}^{(19\times N)}$ is the flattened vector of input *X*, and ${\mathbf{w}}_t \in {\mathbb{R}}^{(19\times N)}$ is the gradient of ${\hat{y}}_t$ at ${\mathbf{r}}_0$. Since each sequence has multiple positive frames, we compute the heatmap ${\mathbf{w}}_H$ by summing ${\mathbf{w}}_t$ at all positive frames,

2 $$\begin{aligned} {\mathbf{w}}_H=\sum _{t\in {\mathcal {P}}}{\mathbf{w}}_{t} = \left. \frac{\partial }{\partial {\mathbf{r}}}\sum _{t\in {\mathcal {P}}}{\hat{y}}_{t}\right| _{{\mathbf{r}}_0} \end{aligned}$$

where ${\mathcal {P}}$ is the set of all the positive frame indices. Each component of ${\mathbf{w}}_H$ indicates the impact (weight) of the corresponding input component of *X* on the positive prediction. Figure 3 shows the visualization of heatmap ${\mathbf{w}}_H$ overlaid on input signal of representative test examples. The second row of Fig. 3 clearly shows the solitary patterns after the band-pass filtering. The gradient-based visualization highlights the solitary region in the input, and qualitatively demonstrates that our model predicts SPs by recognizing toroidally shifted SP patterns.

### Statistical analysis of the boundary burst-SP co-occurrence

**Figure 4:**
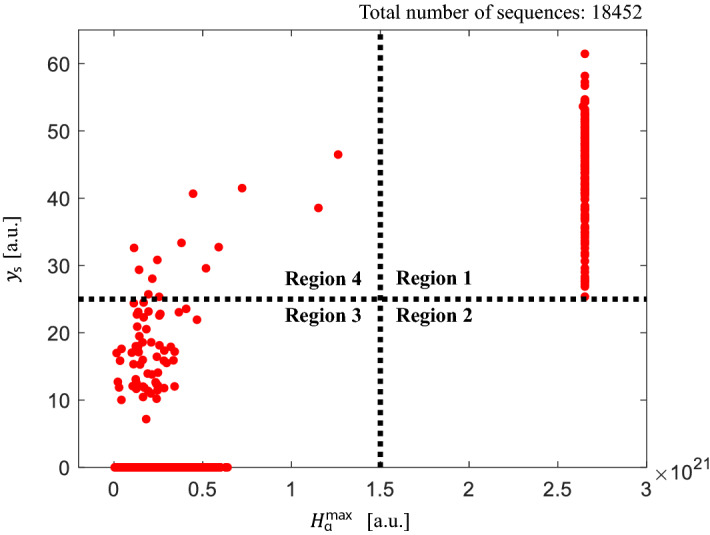
Statistical analysis of boundary burst and SP based on $H_{\alpha }^{\rm{max}}$ and $y_{\rm{s}}$ plot for sequential input data. Regions 1–4 correspond to the cases of burst with SP, burst without SP, no clear burst without SP, and no clear burst with SP, respectively.

Finally, we performed a statistical analysis on the co-occurrences of boundary burst and SPs using a new data set collected from 2018 KSTAR discharges (shots #20540, #20630, #20807, and #21207). This data set is exclusive with the training and test data that were taken from the discharges between 2015 and 2017. The data set consists of a total of 18452 sequences, which are obtained by dividing the data into segments of 400 μs long each. We then labeled each sequence with the existence of a boundary burst and SP. For the burst labeling criterion, we manually inspected the occurrences of burst in the sequences and found that all clear large burst have the maximum H-alpha intensity, $H_\alpha ^{\rm{max}} > 1.5\times 10^{21}$. For the SP labeling, we applied our final model to the individual sequences and obtained the sequence prediction $y_{\rm{s}}$ accumulated over the range of 100 μs centered from the initial high peak as discussed above. We chose $y_{\rm{s}}>25$ as the criterion for the presence of an SP based on our cross-validation with the training data set. Note that, even for an experienced expert, it is still very challenging and time-consuming to manually annotate SPs since it requires choosing a proper bandpass filter and recognizing the SP patterns. Our trained model simplifies this process and allows us to annotate new sequences at no additional human intervention.

Figure 4 plots the values of $H_{\alpha }^{\rm{max}}$ and $y_{\rm{s}}$ to visualize the correlation between the two variables. We identify four regions, and Table 1 summarizes the number of sequences in each region. Region 1 and 2 ascertain whether the boundary burst of high amplitude accompanies SP or not. All the sequences with the clear large burst are in region 1. Region 3 and 4 represent the sequences without a clear signature of boundary burst. Most of these sequences have zero $y_{\rm{s}}$ and are located in region 3. It is worth mentioning that many sequences in region 4 correspond to small boundary burst. Therefore, the most conservative interpretation of the statistical analysis is that a large boundary burst always coexists with SP.

**Table 1:** Number of sequences in each region shown in Fig. 4.

Region	Number of sequences
1 (Burst with SP)	176
2 (Burst without SP)	0
3 (No clear burst without SP)	18,263
4 (No clear burst with SP)	13

## Conclusion

We developed an automatic identification method of SP based on a 2D convolutional deep neural network, and performed a statistical study to follow up on whether SP triggers the boundary burst of magnetized toroidal plasmas^19,24^. The model takes time series data of 19 toroidal MC signals in the KSTAR as inputs and predicts the existence of SPs in each temporal frame. The model was trained in a supervised manner using a data set consisting of real signal manually annotated SPs and synthetic burst signals. Because it is difficult to separate SP and burst signals in the real data using general signal processing methods, we generated synthetic burst data to increase sensitivity to the SP of the developed model. The trained model achieves high performances in three quantitative metrics on a test data set. We also qualitatively confirmed that the output of the model is largely affected by the SP pattern using the gradient-based convolutional neural network visualization technique. Lastly, a statistical analysis of burst-SP co-occurrence was performed using the developed model. This confirmed that a large boundary burst, which is likely to cause severe damage to the fusion device, always involves the emergence of SP.

## Discussion

Our results confirm the universality of the co-occurrence of SP and burst, which reinforces the previous studies that suggested the SP as a candidate for the boundary burst trigger^19,24–26^ . Hence, identifying SP’s generation mechanism will be essential to understand and control the boundary burst of magnetized toroidal plasmas. It will be promising to check the correlation of SP generation with a low-n mode growth through nonlinear interactions of multiple eigenmodes (ELMs)^27–29^ or the growth of a non-normal MHD mode in the presence of a strong flow shear^33^ where normal eigenmodes can no longer exist.

In recent years, deep learning technology has found applications in the field of fusion research and produced meaningful results for the prediction problem of plasma disruption^34,35^. Nonetheless, it remained uncertain whether deep learning techniques can help to study a broader range of physics problems in fusion research such as MHD instabilities. Our result is a meaningful example that illustrates the possibility of applying deep learning techniques for expedient analysis of complex MHD phenomena.

## Methods

### Convolutional neural network

A CNN, which is a commonly used deep neural network for visual image analysis, is built by stacking multiple convolutional and pooling layers. A convolutional layer is a special form of a linear layer where weights (also known as kernels or filters) are restricted to a small adjacent input region^36^. The same kernels are applied to the entire input by shifting over the input regions. Convolutional layers are effective in reducing the number of network parameters and in capturing translation-invariant local patterns over an input region. Note that padding prevents the downsampling of the output by adding dummy values to the border of an input. In our work, to find patterns in a large input patch without downsampling, we instead use dilated convolutional layers. In a dilated convolutional layer, the kernels take an input patch with neighboring values of a finite gap instead of immediate neighboring values. A pooling layer is another most common layer adopted in CNNs. A pooling layer outputs the maximum or average values on the input patch^37^. It generally allows finding patterns in a large input region through downsampling with a large stride (i.e., step size) of the shifting operation. Note that CNNs are the most widely adopted network architecture in computer vision and image processing fields because an CNN architecture can effectively deal with spatial movements of visual objects in images. In our work, the toroidally shifting SP patterns may appear in different temporal and toroidal dimensions for which the transition invariant property of CNNs can be effective.

There are some additional notations that are crucial to note for our network descriptions. A feature map refers to a set of output values of a layer, where each output value is called an activation. Receptive field refers to an input region that affects a particular activation on the network. Activation functions such as sigmoid and rectified linear unit (ReLU) are applied to intermediate feature maps to add nonlinearity to the network. The sigmoid function, $f(x) = 1/(1+e^{-x})$, has a limited output between 0 to 1. Because the gradients in both positive and negative large inputs are close to zero, the sigmoid function has a severe vanishing gradient problem in training using a gradient-based learning method. To overcome this problem, ReLU, which only saturates in the negative direction, is widely used in CNNs^38^.

### Average precision

AP, which is a mean precision over all possible thresholds weighted by recall, is one of the common metric to evaluate the effectiveness of the models. Precision is the ratio of TP to all positive predictions, *i.e.*, ${\rm{TP/(TP+FP)}}$, and recall is the ratio of TP to all actual positives, *i.e.*, ${\rm{TP/(TP+FN)}}$. Here, true (T) and false (F) refer to the correct and incorrect prediction of the model, and positive (P) and negative (N) refer to the presence and absence of an event. For a range of threshold values, precision and recall values are obtained and a precision-recall curve by representing the precision *p*(*r*) as a function of recall *r* can be drawn. If there are multiple precision values for the same *r* value, the maximum precision is taken. Finally, AP is computed by the area under the precision-recall curve:

3 $$\begin{aligned} {\rm{AP}} = \int _{0}^{1} p(r) dr \end{aligned}$$

## Supplementary Information

Supplementary Figures.
