# Supplementary material for: A machine learning approach to identify the universality of solitary perturbations accompanying boundary bursts in magnetized toroidal plasmas
Source: Sci Rep. 2021 Feb 11;11:3662. doi: 10.1038/s41598-021-83192-2 (PMC7878480; doi:10.1038/s41598-021-83192-2)
Supplement: Supplementary file 1 — Supplementary Figures. [file 41598_2021_83192_MOESM1_ESM.pdf]

A machine learning approach to identify the universality of solitary perturbations accompanying boundary bursts in magnetized toroidal plasmas

J. E. Lee[1], P. H. Seo[1], J. G. Bak[2], and G. S. Yun[1*]

[1]*Pohang University of Science and Technology, Pohang 37673, Korea*

[2]*National Fusion Research Institute, Daejeon 34133, Korea*

*Correspondence and requests for materials should be addressed to G.S. Yun (gunsu@postech.ac.kr)

Supplementary Figure.

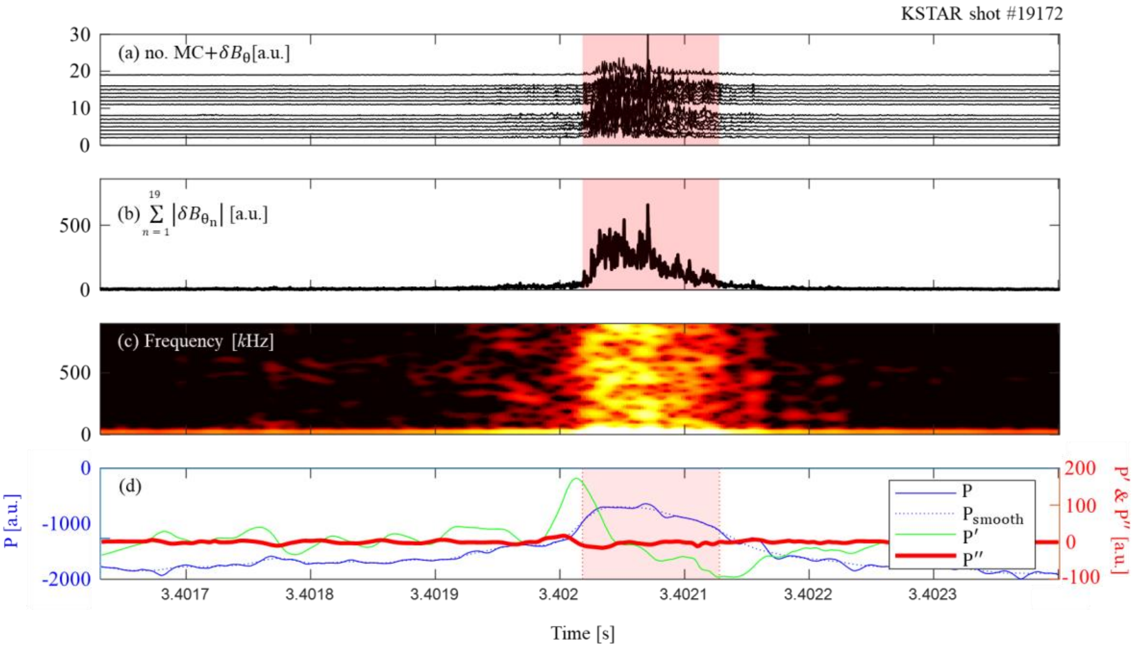

Figure S1. Determination of the burst duration (KSTAR shot #19172). (a) absolute value of 19 toroidal MC signals, (b) summation of the absolute of 19 MC signals, (c) spectrogram of the signal in (b) using FFT with a window length of 64 and overlap of 95 %, and (d) blue, green and red respectively indicate the sum of the spectral power density of all frequencies for each FFT execution time range (P), the first derivative of P (P'), and the second derivative of P (P"). The burst start time $\boldsymbol{t_{\mathrm{st}}}$ is $\boldsymbol{t_{\mathrm{ref}} + 16\ \mu s}$ where $\boldsymbol{t_{\mathrm{ref}}}$ is the max of P" near the max of P' and $\boldsymbol{16\ \mu s}$ corresponds the time of half of FFT window size. The burst finish time $\boldsymbol{t_{\mathrm{fh}}}$ is first-time point where P is less than $\mathbf{P}_{\boldsymbol{t_{\mathrm{st}}}}$ after $\boldsymbol{t_{\mathrm{st}}}$ minus $\boldsymbol{16\ \mu s}$. The burst duration $\boldsymbol{t_{\mathrm{fh}} - t_{\mathrm{st}}}$ is highlighted in red.

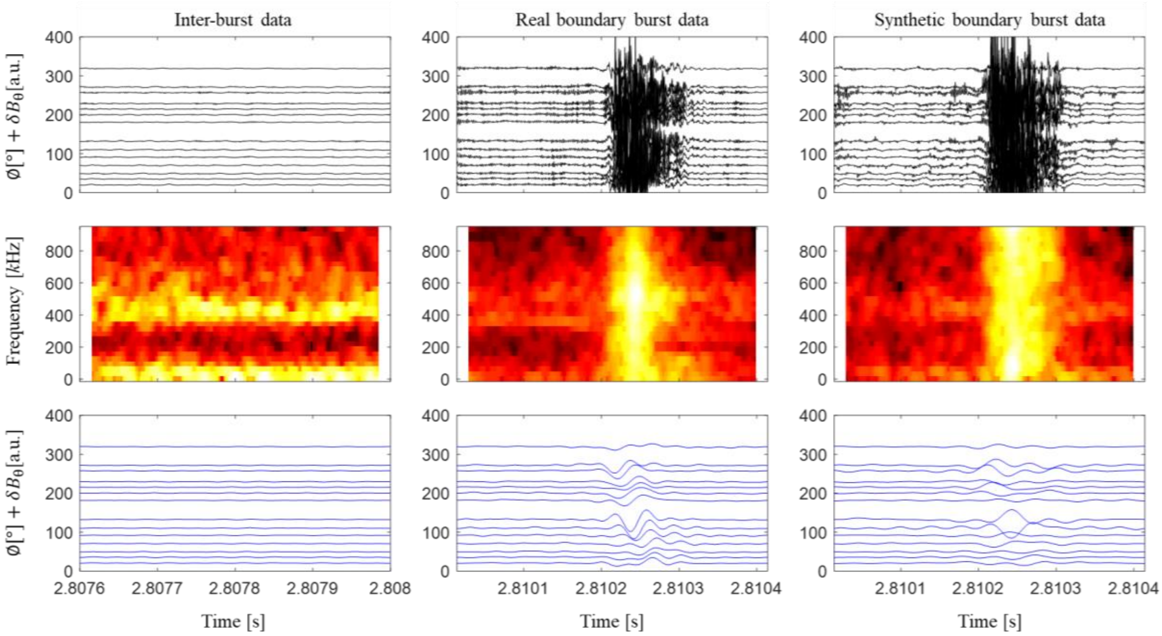

Figure S2. Comparison among real data in the inter-burst period, real boundary burst data, and synthetic boundary burst data. The first rows represent the raw MC signals. The synthetic data are generated by multiplying the envelope of the real MC signals with a white noise. The second rows show the spectrogram of the raw signals of the $\mathbf{11^{th}}$ MC channel. The third rows show the bandpass filtered (5 to 30 $\boldsymbol{k}\mathbf{Hz}$) data. The synthetic burst signal has a considerably strong broadband spectrum but no low frequency phase correlation between adjacent toroidal MC channels.
